# Health-related quality of life in children with presumptive TB

**DOI:** 10.5588/ijtldopen.24.0355

**Published:** 2024-11-01

**Authors:** M.G. Anthony, L.S. Johnson, M. van Niekerk, A. Mfwaze, B. Bavuma, A.C. Hesseling, G. Hoddinott, M.M. van der Zalm

**Affiliations:** ^1^ Desmond Tutu TB Centre, Department of Paediatrics and Child Health, Faculty of Medicine and Health Sciences, Stellenbosch University, Cape Town, South Africa;; ^2^ Program in Public Health, Feinberg School of Medicine, Northwestern University, Chicago, IL, USA;; ^3^ School of Public Health, Faculty of Medicine and Health, The University of Sydney, Sydney, NSW, Australia.

**Keywords:** presumptive tuberculosis, health-related quality of life, TANDI, EQ-5D-Y

## Abstract

**BACKGROUND:**

Respiratory illnesses, including pulmonary TB (PTB), cause significant morbidity. We aimed to understand the health-related quality of life (HRQoL) of children with presumptive PTB.

**METHODS:**

Children aged 0–13 years presenting with presumptive PTB were enrolled. This study includes children who started TB treatment and children in whom TB was excluded (symptomatic controls). Quantitative data were collected using the Toddler and Infant quality of life Instrument (TANDI) (<3 years) and European Quality of Life-5 Dimensions-Youth (EQ-5D-Y) (>3 years) measures. Qualitative data were collected through in-depth interviews using thematic analysis.

**RESULTS:**

Quantitative data from caregivers of 201 children (TANDI: *n* = 170; EQ-5D-Y: *n* = 31) showed 77 (38.3%) were diagnosed with TB, while 124 (61.7%) were symptomatic controls. Qualitative data from 15 caregivers of 21 children included 10 (67%) children with TB and 5 (33%) symptomatic controls. The median TANDI Visual Analogue Score (VAS) for overall health was 90% (IQR 80–100); the EQ-5D-Y VAS median was 95% (IQR 80–100). Caregivers described decreased energy, difficulty eating, and increased sleep using qualitative interviews, which were not reflected in the quantitative data. No differences were found between children with TB and symptomatic controls.

**CONCLUSIONS:**

HRQoL was high in children with TB, but discrepancies between quantitative and qualitative measures highlight the limitations of the current HRQoL measures.

## RÉSUMÉ

**CONTEXTE:**

Les affections respiratoires, notamment la TB pulmonaire (PTB), engendrent une morbidité significative. Nous avons entrepris d'explorer la qualité de vie liée à la santé (HRQoL) des enfants présentant une TB présumée.

**MÉTHODES:**

Des enfants âgés de 0 à 13 ans, suspectés de PTB, ont été sélectionnés pour cette étude. Celle-ci comprend à la fois les enfants ayant débuté un traitement contre la TB et ceux chez qui la maladie a été écartée, considérés comme témoins symptomatiques. Les données quantitatives ont été obtenues grâce à l'outil d'évaluation de la qualité de vie des nourrissons et des jeunes enfants (TANDI, pour l’anglais « *Toddler and Infant quality of life Instrument* ») (<3 ans), ainsi qu'à l'outil d'évaluation de la qualité de vie européenne à 5 dimensions pour les jeunes (EQ-5D-Y, pour l’anglais « *European Quality of Life-5 Dimensions-Youth* ») (>3 ans). Par ailleurs, des données qualitatives ont été recueillies par le biais d'entretiens approfondis, suivis d'une analyse thématique.

**RÉSULTATS:**

Les données quantitatives obtenues auprès des soignants de 201 enfants (TANDI : *n* = 170 ; EQ-5D-Y : *n* = 31) ont révélé que 77 enfants (38,3%) avaient reçu un diagnostic de TB, tandis que 124 enfants (61,7%) étaient des témoins présentant des symptômes. Les informations qualitatives recueillies auprès de 15 soignants de 21 enfants ont montré que 10 enfants (67%) étaient atteints de TB et 5 enfants (33%) étaient des témoins symptomatiques. La médiane de l'échelle visuelle analogique (VAS, pour l’anglais « *Visual Analogue Score* ») pour l'état de santé général selon le TANDI était de 90% (IQR 80–100), tandis que la médiane du VAS pour l'EQ-5D-Y était de 95% (IQR 80–100). Lors des entretiens qualitatifs, les soignants ont mentionné une diminution de l'énergie, des difficultés alimentaires et une augmentation du sommeil, des éléments qui n'ont pas été reflétés dans les données quantitatives. Aucune différence significative n'a été observée entre les enfants atteints de TB et les témoins symptomatiques.

**CONCLUSIONS:**

La HRQoL était significativement élevée chez les enfants atteints de TB, cependant, les différences observées entre les évaluations quantitatives et qualitatives mettent en évidence les insuffisances des outils de mesure actuels de la HRQoL.

Respiratory illnesses in children <5 years old are a major global health issue, causing approximately five million deaths annually, with over 90% occurring in low- and middle-income countries (LMICs).^1,2^ South Africa, facing significant challenges, sees acute respiratory infections (ARIs) contributing to over 6% of the global disease burden, primarily in children <5 years in LMICs.^3^ PTB is a significant cause of respiratory illness in LMICs, with around 1.2 million new TB cases and 214,000 deaths among children aged <14 years in 2022.^4^

PTB is often clinically indistinguishable from other common childhood respiratory illnesses, especially in children aged <5 years,^5,6^ with few children being microbiologically confirmed for TB. While most data on the impact of disease and treatment focus on adults, evidence suggests TB also affects health-related quality of life (HRQoL) and lung health in children and adolescents.^7–11^ For instance, a study on children with PTB revealed a notable decline in HRQoL post-treatment.^7^ Children with TB may experience decreased energy and sleep disturbances due to symptoms and stigma.^11^

HRQoL is crucial for children aged <5 years, encompassing physical health, emotional well-being, and social interactions. Despite the high burden, data on childhood TB morbidity and psychosocial impacts are limited. Data are available on the morbidity of childhood TB and even the psychosocial consequences of TB.^12,13^

To address this gap, we conducted a mixed-method study to understand HRQoL in children with presumptive TB. We used the Toddler and Infant Quality of Life Instrument (TANDI) and European Quality of Life-5 Dimensions-Youth (EQ-5D-Y) tools for quantitative measurements and conducted in-depth interviews with a sub-sample to explore their HRQoL experiences.^14–17^

## METHODS

### Study design

This study used a mixed-method concurrent parallel design and collected data within a prospective observational TB diagnostic cohort study called Umoya, which has been previously described.^18^

### Study participants and setting

Children with presumptive TB at the Karl Bremer Hospital (KBH) and Tygerberg Hospital (TBH) in the Cape Metropolitan District (CMD) were enrolled between March 2021 and December 2023.^18^ The TB incidence among young children (0–5 years) in Cape Town, South Africa, is ∼511 per 100,000.^19^ These hospitals serve areas with significant socioeconomic challenges including unemployment, poverty, and substance abuse are prevalent. These conditions contribute to poor nutrition, pollution, and increased HIV exposure, heightening the risk of respiratory illnesses and TB.^20,21^ Although hospital-referred children are generally considered more ill in childhood TB, especially for children aged <5 years, primary health clinics refer them for TB sampling or treatment decisions. Hospital physicians, independent of the study team, made treatment decisions. Children who began TB treatment had clinically or microbiologically confirmed TB, while those not started on treatment were classified as ‘symptomatic controls’. HRQoL assessments were conducted during routine clinical visits.

### Data collection procedures

#### Quantitative data collection

We used two generic HQROL instruments tailored to different age groups: TANDI for children aged <3 years, to measure movement, play, pain, relationships, communication, and eating;^22^ and the EQ-5D-Y was for children aged >3 years, measuring mobility, self-care, usual activities, pain/discomfort, and emotional well-being.^23^ Both instruments included a visual analogue scale (VAS) for general health assessment from 0 (worst) to 100 (best).^22,23^ Data were captured via electronic case report forms into the REDCap (Research Electronic Data Capture) database (Vanderbilt University, Nashville, TN, USA).^24^

#### Qualitative data collection

A purposive sub-sample of Umoya cohort participants was selected for in-depth qualitative interview, conducted iteratively until theoretical saturation. Interviews were held at baseline (July–October 2021), and vignettes and participatory activities were used to explore children's experiences. Conducted in the caregivers' preferred language (English, Xhosa, or Afrikaans), the interviews were audio-recorded with consent and lasted 45–60 min. Data collectors (MA, TM, AM) wrote semi-structured case summaries, which senior researchers (GH, MMvdZ) reviewed for analytic rigour.

### Data analysis

#### Quantitative data analysis

Data were exported to SPSS v29.0.1.0 (IBM Corp, Armonk, NY, USA) for statistical analysis using descriptive statistics.

#### Qualitative data analysis

Verbatim transcriptions and translations were analysed thematically to identify the key experiences of each participant from the semi-structured interviews.^25,26^

### Ethics considerations

The Umoya study, including the HRQoL assessments and interviews, was approved by the Health Research Ethics Committee (HREC) at the Stellenbosch University, Tygerberg, South Africa (N17/08/083 & S21/05/084). Caregiver participants provided written informed consent at recruitment.

## RESULTS

### Characteristics of the sample

#### Quantitative component

At baseline, 201 questionnaires were completed, with TANDI (*n* = 170) and EQ-5D-Y (*n* = 31) (Table 1). The median age of children in this sub-sample was 2.3 years old (interquartile range [IQR] 0.8–3.3), with an equal sex distribution. Of these children, 77 (38.3%) were diagnosed with TB, and 124 children (61.7%) were symptomatic controls. Human immunodeficiency virus (HIV) exposure was noted in 59 (29.4%) children, with 12 (6.0%) living with HIV.

**Table 1.** Baseline demographic characteristics of children by age group.

	All participants (*n* = 201) *n* (%)	TANDI (age 0–<3 years) (*n* = 170) *n* (%)	EQ-5D-Y (age 4–<10 years) (*n* = 31) *n* (%)
*Sex*			
Male	93 (46.3)	80 (47.1)	13 (41.9)
Female	108 (53.7)	90 (52.9)	18 (58.1)
Age, years, median [IQR]	2.3 [0.8–3.3]	1.4 [0.7–2.6]	5.5 [5.5–6.7]
Classification			
Children with TB	77 (38.3)	69 (40.6)	8 (25.8)
Symptomatic children	124 (61,7)	101 (59.4)	23 (74.2)
HIV exposure			
Yes	59 (29.4)	52 (30.6)	7 (22.6)
No	140 (69.7)	117 (68.8)	23 (74.2)
Unknown	2 (1)	1 (0.6)	1 (3.2)
Living with HIV			
Yes	12 (6)	9 (5.3)	3 (9.7)
No	201 (92.5)	158 (92.9)	28 (90.3)
Unknown	3 (1.5)	3 (1.8)	0 (0)

TANDI = Toddler and Infant health-related quality of life instrument; EQ-5D-Y= European Quality of Life-5 Dimensions-Youth; IQR = interquartile range.

#### Qualitative component

The qualitative sample included 15 caregivers of 21 children with four sibling pairs. In 3 pairs, both children were diagnosed with TB, while in 1 pair, one child had TB, and the other was a symptomatic control. One caregiver had three children in the study, all of whom were symptomatic controls. Ages ranged from 3 months to 5 years, with 6 (31.6%) girls and 13 (68.4%) boys. Of 21 children, 15 (71.4%) children were diagnosed with PTB, and 6 children were symptomatic controls (28.6%). Of the 15 caregivers, 11 (73.3%) reported having a household TB contact.

### TANDI findings (ages 0–3 years)

Most caregivers reported no problems across HRQoL domains (Table 2). The median TANDI VAS describing participants' general health was 90% with an IQR of 80–100%. Notably, 54 (31.8%) rated their children’s health as 100% (Table 3) and only (0.6%) reported health below 50%.

**Table 2.** Caregiver proxy report of the TANDI quality of life of children at baseline.

Dimension	Level	Children with presumptive TB (*n* = 170) *n* (%)
Movement	No problems	163 (95.9)
	Some problems	6 (3.5)
	A lot of problems	1 (0.6)
Play	No problems	161 (94.7)
	Some problems	9 (5.3)
	A lot of problems	0 (0)
Pain	No problems	132 (77.6)
	Some problems	37 (21.8)
	A lot of problems	1 (0.6)
Relationships	No problems	164 (96.5)
	Some problems	6 (3.5)
	A lot of problems	0 (0)
Communication	No problems	146 (85.9)
	Some problems	24 (14.1)
	A lot of problems	0 (0)
Eating	No problems	136 (80.0)
	Some problems	28 (16.5)
	A lot of problems	5 (2.9)

TANDI = Toddler and Infant health-related quality of life instrument.

**Table 3.** Caregiver proxy report of children’s EQ-5D-Y quality of life at baseline by illness category.

Dimension	Level	Children with presumptive TB (*n* = 31) *n* (%)
Mobility	No problems	31 (100.0)
	Some problems	0 (0)
	A lot of problems	0 (0)
Looking after himself/herself	No problems	25 (80.6)
	Some problems	6 (19.4)
	A lot of problems	0 (0)
Doing usual activities	No problems	28 (90.3)
	Some problems	3 (9.7)
	A lot of problems	0 (0)
Having pain or discomfort	No problems	26 (83.9)
	Some problems	5 (16.1)
	A lot of problems	0 (0)
Feeling worried, sad or unhappy	No problems	27 (87.1)
	Some problems	4 (12.9)
	A lot of problems	0 (0)

EQ-5D-Y= European Quality of Life-5 Dimensions-Youth.

However, qualitative interviews highlighted significant concerns about mobility and energy levels, which were less apparent in quantitative data. For example, a mother of a 3-year-old girl and a 5-year-old boy diagnosed with TB noted severe fatigue and lack of activity:

They just wanted to sleep and were nagging. They also did not want to eat. They were not active; they were not like children that would play […] The children looked dead; they were just lying there, they never sat up in the bed and played.

Pain was reported in 37 (21.8%) of children, consistent with qualitative findings such as a mother describing her child’s cough and pain. The mother of a 3-year-old boy diagnosed with TB reported:

My child was coughing, the body temperature was high, and he was in pain.

While 6 (3.5%) of children reported relationship problems and 9(5.3%) had problems with play. Interviews revealed that reduced playfulness often signalled distress rather than direct communication problems. For instance, a mother of a 3-year-old boy observed a marked decrease in playfulness during illness. She said:

He was always playful and energetic, but when he was sick, he stopped playing and was always sleeping; even when he was trying to play, he would quickly sit down.

Eating problems were reported in 28 (16.5%) of children, with 5 (2.9%) experiencing significant problems. These were confirmed by interviews including a mother of a 1-year-old boy diagnosed with TB who noted her child’s loss of appetite and weight:

He lost weight and appetite; I notice when he is not eating that there is a problem because he likes food.

### EQ-5D-Y findings (age >4 years)

Most caregivers reported no problems across EQ-5D-Y domains (Table 3). The median for EQ-5D-Y VAS was 95% (IQR 80–100%). Nearly half (48.4%) rated their children’s health 100%, but 3 (9.7%) rated it below 60%. Qualitative interviews revealed mobility challenges, with a mother noting her 4-year-old child with TB stayed indoors and did not play with friends:

When she was sick, she did not play with her friends; she was just in the house.

While 6 (19.4%) of children had self-care issues in the quantitative survey, none were reported in qualitative interviews. Some caregivers 3 (9.7%) reported declines in usual activities, with children preferring rest. A caregiver of a 3-year-old girl and 5-year-old boy diagnosed with TB:

Yes, they just wanted to lay [..] they were not active.

Pain and discomfort were noted in 5 (16.1%) of children, often linked to emotional distress, as seen in a mother’s account of her child’s crying due to pain. A mother of a 4-year-old girl diagnosed with TB reported:

She was a bit difficult because she could not breastfeed. I wished that I could take the pain away for her, but I couldn’t; she was just crying.

Additionally, 4 (12.9%) experienced feelings of worry or sadness, with caregivers noting stigma and distress from TB-related physical changes such as weight loss. A mother of a 5-year-old boy diagnosed with TB:

Nathan didn’t want to play with the other children anymore, and he was very shy because he was very thin [lost weight due to TB]. He always told me that he does, and they [friends] would laugh at him because he is so thin.

### Qualitative insights into additional HRQoL impacts beyond TANDI and EQ-5DY

Caregivers highlighted several key factors affecting their children’s HRQoL, focusing on emotional well-being, behavioural changes, social well-being, and early development. Emotional distress was a significant concern, with caregivers reporting increased crying and fear, particularly during treatments. For example, a caregiver of a 2-year-old girl and a 1-year-old boy diagnosed with TB reported:

She cries a lot in general; however, he will cry a lot more when he is sick because he can’t talk, so he will cry a lot.

A mother of a 4-year-old boy reported:

He used to cry and run away when he had to take his pills. When he has to go to the clinic, he would cry; I think it is because he did not want to take those pills or do not want to test.

Behavioural changes, like clinginess and withdrawal, were also common but were not typically captured by standard HRQoL measures. A mother of a 1-year-old noted how her child became withdrawn:

There are times that she will isolate herself and just want to play alone and so on, or she does not want to play at all. She also does not want to go to anyone else, and she just wants to be in my arms.

Social support was vital, with caregivers relying on family and community during their child’s illness. One caregiver shared how her family helped with hospital visits and care. A caregiver of a 2-year-old reported:

All the family knew about the child’s condition […] they were supportive because they took us to hospital and my family visited my child was in the hospital and they came to fetch us when we were discharged from hospital.

Developmental impacts were another concern, with caregivers observing delays in milestones and disruptions in schooling. For instance, a mother had to withdraw her 3-year-old from crèche to focus on treatment, and others noted delays in speech and motor skills linked to illnesses. A mother of a 3-year-old boy reported:

I had to stop him from going to crèche so that he can focus on taking treatment and went back again after he had finished his treatment.

A caregiver of a 3-year-old boy said:

He could not talk properly when he was 2 years old he would fall around a lot [when walking] […] He was not like my children when I look at how my children were at that stage. He was very behind in those aspects, and I don’t know if it was because of the illness.

In summary, these findings showed that caregivers tend to underreport the changes in their children when standard tools are used, but they can articulate a detailed description of problems that could map onto several domains.

## DISCUSSION

Our study found that HRQoL, as measured by standard tools, appeared high among children despite caregivers reporting significant challenges in qualitative interviews. This discrepancy suggests that existing HRQoL tools may not fully capture the complexities faced by children, particularly those evaluated for TB and other respiratory illnesses.

The high HRQoL scores in our study align with other research, where 58-88% of participants reported no issues across six HRQoL dimensions despite having acute or chronic conditions. Consistent with previous studies in infants and toddlers with conditions like asthma, diabetes, cerebral palsy, respiratory illnesses, and burn injuries, we found that pain and eating were the most affected dimensions.^14,15,27^ These studies also found pain and discomfort to be the most important dimensions for children and eating problems to be the second most frequently reported problem.^14,27^

The low reported frequencies of HRQoL problems hint at a ceiling effect for a standardised instrument like the EQ-5D-Y, particularly in children who may have adapted to their illness or treatment routines.^28,29^ The ceiling effect occurs when scores tend to cluster near the highest possible score on a quality of life (QoL) measure. This can make detecting subtle differences or improvements difficult because the tool essentially ‘runs out of room’ to capture higher or more varied responses.^30^ Another explanation for these low problem frequencies and high ratings of HRQoL could be necessitated coping, meaning that the children and caregivers have learned to live with the disease and do not perceive the limitations to be as bad as expected.^31^

Standardised tools might not fully capture the range of experiences, leading to underreporting of problems. In open-ended interviews, participants can express concerns more freely, which might reveal issues missed in structured questionnaires. Social desirability bias could also vary; participants may respond differently to a clinical assessor in quantitative settings than a confidant in qualitative interviews. The qualitative process fosters rapport, gradually easing participants' guardedness with non-judgmental and encouraging feedback. Given these limitations, it is crucial to consider what can be done for children currently affected by TB and other respiratory illnesses. While more research and better tools are needed, immediate steps can be taken using the methodologies employed in our qualitative data collection. These tools, which include in-depth interviews and caregiver reports, provide a more nuanced understanding of children’s experiences and could be integrated into routine clinical assessments to uncover challenges that standard tools might miss.

For instance, qualitative approaches could be used alongside existing HRQoL measures to better understand the specific domains of behaviour, early development, and social well-being that caregivers identify as important. This hybrid approach could provide a more comprehensive assessment of a child’s HRQoL, guiding more personalised care and interventions. A limitation of including a generally sicker population in our study is that it may restrict the generalisability of the findings to children with less severe conditions, potentially skewing the HRQoL outcomes toward more severe symptomatology.

A major strength of our study was the comprehensive assessment of HRQoL in young children with and without TB, which allowed us to identify critical discrepancies between quantitative measures and qualitative insights. However, the small number of completed EQ-5D-Y questionnaires and the focus on a single geographical area may limit the generalizability of our findings.

Despite these limitations, our findings offer valuable perspectives on the HRQoL of young children in a context with a high burden of TB, highlighting the need for improved HRQoL measures that are more responsive to the complexities of childhood illness in LMICs. By refining HRQoL domains, we can better evaluate the impact of TB on young children’s lives and develop strategies to enhance their well-being and that of their families.

There is an ethical imperative to ensure that the challenges faced by children with TB are recognised and addressed, even if current tools fall short. Rather than abandoning the measurement of HRQoL in this population, there is a need to refine and expand the domains included in these assessments. This could involve developing new tools or adapting existing ones to better capture the lived experiences of children in LMICs.

Our study underscores the importance of refining HRQoL measures to include dimensions such as behaviour, early development, and physical health beyond basic mobility. By doing so, we can more accurately assess the impact of TB and other respiratory illnesses on children’s quality of life and inform interventions that address their unique needs.
